# Nitrogen addition alters plant growth in China’s Yellow River Delta coastal wetland through direct and indirect effects

**DOI:** 10.3389/fpls.2022.1016949

**Published:** 2022-10-13

**Authors:** Liwen Zhang, Lianjun Zhao, Huapeng Yi, Siqun Lan, Lin Chen, Guangxuan Han

**Affiliations:** ^1^ Tianjin Key Laboratory of Animal and Plant Resistance, College of Life Sciences, Tianjin Normal University, Tianjin, China; ^2^ Chinese Academy of Sciences (CAS) Key Laboratory of Coastal Environmental Processes and Ecological Remediation, Yantai Institute of Coastal Zone Research (YIC), Chinese Academy of Sciences (CAS) Shandong Key Laboratory of Coastal Environmental Processes, YICCAS Yellow River Delta Field Observation and Research Station of Coastal Wetland Ecosystem, YICCAS, Yantai, China; ^3^ School of Resources and Environmental Engineering, Ludong University, Yantai, China; ^4^ College of Environment and Planning, Liaocheng University, Liaocheng, China

**Keywords:** nitrogen addition, coastal wetland, soil properties, bacterial diversity, plant traits, SEM

## Abstract

In the coastal wetland, nitrogen is a limiting element for plant growth and reproduction. However, nitrogen inputs increase annually due to the rise in nitrogen emissions from human activity in coastal wetlands. Nitrogen additions may alter the coastal wetlands’ soil properties, bacterial compositions, and plant growth. The majority of nitrogen addition studies, however, are conducted in grasslands and forests, and the relationship between soil properties, bacterial compositions, and plant growth driven by nitrogen addition is poorly understood in coastal marshes. We conducted an experiment involving nitrogen addition in the *Phragmites australis* population of the tidal marsh of the Yellow River Delta. Since 2017, four nitrogen addition levels (N0:0 g • m^-2^ • year^-1^, N1:5 g • m^-2^ • year^-1^, N2:20 g • m^-2^ • year^-1^, N3:50 g • m^-2^ • year^-1^) have been established in the experiment. From 2017 to 2020, we examined soil properties and plant traits. In 2018, we also measured soil bacterial composition. We analyzed the effect of nitrogen addition on soil properties, plant growth, reproduction, and plant nutrients using linear mixed-effect models. Moreover, structural equation modeling (SEM) was utilized to determine the direct and indirect effects of nitrogen addition, soil properties, and bacterial diversity on plant growth. The results demonstrated that nitrogen addition significantly affected plant traits of *P. australis*. N1 and N2 levels generally resulted in higher plant height, diameter, leaf length, leaf breadth, and leaf TC than N0 and N3 levels. Nitrogen addition had significantly impacted soil properties, including pH, salinity, soil TC, and soil TS. The SEM revealed that nitrogen addition had a direct and positive influence on plant height. By modifying soil bacterial diversity, nitrogen addition also had an small indirect and positive impact on plant height. However, nitrogen addition had a great negative indirect impact on plant height through altering soil properties. Thus, nitrogen inputs may directly enhance the growth of *P. australis* at N1 and N2 levels. Nonetheless, the maximum nitrogen addition (N3) may impede *P. australis* growth by reducing soil pH. Therefore, to conserve the coastal tidal marsh, it is recommended that an excess of nitrogen input be regulated.

## Introduction

Agriculture and industrialization are major contributors to the global release of reactive nitrogen into the environment. For example, the utilization of nitrogen fertilizer in agriculture increased from 12 Mt in 1961 to 110 Mt in 2014, and a considerable proportion of nitrogen inputs ended up in terrestrial and aquatic ecosystems, resulting nitrogen enrichment and contamination (Bodirsky et al., 2014; Yu et al., 2019). Then, nitrogen overloading may alter soil properties, soil microorganisms, and plant compositions, including species richness, and abundance, leading to a shift in ecosystem functions (He et al., 2021a; Zhong et al., 2022). These consequences will challenge ecosystem conservation and management. Therefore, simulative nitrogen addition studies have been done in numerous ecosystems, and outcomes from these experiments can shed light on the consequences and mechanisms of nitrogen enrichment on ecosystems.

Nitrogen enrichment can directly and substantially affect soil physical and chemical properties. The experiments focus primarily on measuring soil pH, which is a crucial and comprehensive component. The soil pH responds sensitively to nitrogen enrichment, particularly in acidic soils. Nitrogen addition can reduce soil pH, because NH_4_^+^ is coupled with OH^-^ in the soil and then released into the air as NH_3_; as a result of the depletion of OH^-^ in the ground, the pH value decreases, and the earth becomes more acid (Zhou et al., 2017). The drop of soil pH following nitrogen addition caused variations in soil enzyme activity (Liu and Zhang, 2019), microbial communities (Zhou et al., 2017), and plant community composition (Liu et al., 2020). In addition, an increase in nitrogen input resulted in stoichiometric imbalances (Li et al., 2016). For instance, nitrogen addition increased the amount of available N (AN) but lowered the amount of soil available P (AP) in the soil (Touhami et al., 2022), which might increase the N:P ratio in the soil and hinder the growth of microorganisms or plants. Yuan et al. (2019) reported that in the non-linear relationship between nitrogen addition and C:N ratio, C:P ratio, and N:P ratio, the greatest stochiometric imbalance occurred at the intermediate nitrogen addition level, which promoted the microbial C limitation but not P limitation.

Nitrogen addition has detrimental effects on the microbial community. For example, nitrogen addition decreased soil fungal richness (Leff et al., 2015; Yuan et al., 2020) and soil bacterial diversity (Lu et al., 2021). Furthermore, soil acidification and the modification of resources by nitrogen addition are recognized as the primary mechanisms underlying the diversity in microbial communities. Chen et al. (2015) demonstrated that nitrogen-induced soil acidification was the primary factor inhibiting bacterial, fungal and actinobacteria biomass. In a long-term nitrogen addition experiment, Liu et al. (2020) also revealed that the decrease in soil pH was attributable to the loss of soil bacterial diversity. However, Zhou et al. (2017) demonstrated through a meta-analysis that nitrogen addition promoted the increase of resources, whereas soil acidification had no effect on microbial communities.

Depending on the amount of nitrogen added, nitrogen addition has both beneficial and negative impacts on plant growth and community productivity. High and abrupt nitrogen addition was toxic for plant growth (Gao et al., 2014) whereas, intermediate nitrogen addition increased leaf nutrients, total leaf biomass (Liang et al., 2020), and carbon in plant shoots (Sun et al., 2020; Wang et al., 2020), seedling performance (Shi et al., 2020), and the stability of aboveground net primary productivity plant community (Chen et al., 2016b; Yang et al., 2022).

Few studies have investigated the effects of nitrogen enrichment on coastal wetland ecosystems, whereas numerous studies have examined the effects of nitrogen enrichment on grasslands and forests (Zhou et al., 2017). Coastal regions account for only 4% of the worldwide geographical area, although they are home to one-third of the global population. Diversity of fish, birds, and benthic animals inhabit coastal wetlands. Coastal wetlands also provide human with numerous ecosystem services, such as carbon sequestration, storm mitigation, and coastline protection. Similar to other ecosystems, nitrogen is a limiting nutrient for plant growth in coastal wetlands, despite the considerable nitrogen storage capacity of coastal wetland. Thus, the coastal wetland often was frequently utilized as a buffer zone or reservoir for the removal excess nitrogen from agricultural lands prior to its discharge into the ocean. Empirical research indicating that the excessive nitrogen inputs may severely impair the coastal wetland vegetation (Deegan et al., 2012), suggesting that the vegetation of the coastal wetland is susceptible to nitrogen fluctuations. Contradictory evident indicated that 13-year nutrient enrichment did not affect the ecosystem stability (e.g., soil strength and structural integrity of the soil matrix) in the coastal marsh, whereas the belowground root biomass was reduced in the coastal marsh (Graham and Mendelssohn, 2014). Therefore, further research is required to determine the impact of nitrogen enrichment on soil properties and vegetation growth in the coastal wetlands. Furthermore, the linkage among nitrogen addition, soil properties, bacterial composition, and plant growth in the coastal wetland could be established. In this study, we addressed the following scientific question: how do soil properties, bacterial composition, and plant growth respond to nitrogen addition in the coastal wetland? This study will deepen our understanding of the effect and the underlying mechanism of nitrogen addition on plant growth in the coastal wetland. It also will provide suggestions on the conservation of coastal wetlands in the context of environmental change.

## Methods

### Study site

The study site was located in the salt marsh of the Yellow River Delta (YRD) in Shandong Province (37°44’5’’ N, 119°12’56’’ E). The climate of this region is warm temperate, and the annual average temperature is 11.7-12.6°C. The average yearly precipitation is 530-630 mm, and most of the precipitation occurs in July and August. The average yearly evaporation is 1750-2430 mm, and tidal flooding occurs through irregular semilunar and semidiurnal tides. In the YRD, the dominant native species are *P. australis* and *Suaeda salsa*; and *P. australis* distribute in the high marsh while *S. salsa* dominates the low marsh. During the growth season (May to November), the total N deposition rate in the YRD was estimated to be around 22.64 kg/hm^2^ (Guan et al., 2019).

### Field experiments

In 2017, the experiment plots were randomly established in the *P. australis* vegetation of the high marsh; the nitrogen addition levels were 0 g • m^-2^ • year^-1^ (N0), 5 g • m^-2^ • year^-1^ (N1), 20 g • m^-2^ • year^-1^ (N2), and 50 g • m^-2^ • year^-1^ (N3) (Zhao et al., 2021). Urea [CO (NH_2_)_2_ containing 46% nitrogen] was added in May and July each year and it was scattered evenly in the plot. Each level had six replicates. The plot size was 2 × 2 m^2^. The distance between the two plots was greater than 10 m. The height of *P. australis* individuals in the initial plots was similar in May 2017. During 2017 and 2020, the diameter, height, leaf numbers, leaf length, and leaf breadth of each individual, and the total individuals in the plot were measured in the middle of May, Jul., and Sep. In Sep. 2017~2020, the soil at a depth of 0~10 cm and mature leaves from each plot were sampled. One portion of the fresh soil was refrigerated at -20°C and then used to determine the NO_3_-N, and NH_4_-N contents, the other portion of the soil was air-dried to measure soil pH, electrical conductivity (EC), total C (TC), total N (TN), total P (TP), AP, and total S (TS). In Sep. 2018, one portion of the fresh soil was frozen at -80°C, and utilized to determine the bacterial composition. In addition, the leaves were collected to determine leaf nutrients, including TC, TN, TP, and TS.

### Soil and plant analysis

The elemental analyzer (Vario Micro cube, Elementar Co., Germany) was employed to measure TC and TN in soil and leaves. The pH meter (QT-PH220S, Beijing Channel Scientific Instrument Co., Ltd, China) was used to determine soil pH (soil: water = 1: 5). The continuous flow analyzer (AutoAnalyzer III, Seal Co., Germany) was used to measure NO_3_-N and NH_4_-N contents [extraction: 3 g of fresh soil using a 15 ml KCl solution (2 mol/L)]. The total amount of AN was the sum of NO_3_-N and NH_4_-N contents. The ultraviolet spectrophotometer (T6NewCentury, Beijing Persee General Instrument Co., Ltd, China) was employed to determine TP and AP content by the Oslen method.

### Soil bacterial composition

The soil bacterial diversity was determined by 16S rRNA. 0.5 g of soil was used to extract DNA. The genomic DNA of the sample was extracted by the CTAB method. Then, the purity and concentration of DNA were detected by agarose gel electrophoresis. A suitable sample of DNA was used in a centrifuge tube and diluted with sterile water to 1 ng/μl. Using the diluted genomic DNA as the template and according to the selection of sequencing region, the specific primers with barcodes were used. Phusion^®^ High-Fidelity PCR Master Mix with GC Buffer and high efficiency and high-fidelity enzyme were used for PCR to ensure amplification efficiency and accuracy. PCR products were detected by electrophoresis with agarose gel of 2% concentration. According to the concentration of PCR products, the samples were mixed equally, and then the PCR products were detected by 2% agarose gel electrophoresis. The target bands were recovered from the gel recovery kit provided by the Qiagen company. Truseq ^®^ DNA PCR Free Sample Preparation Kit was used for library construction. The constructed library was quantified by Qubit and Q-PCR. After the library was qualified, NovaSeq6000 was used for computer sequencing. According to the Barcode sequence and PCR amplified primer sequence, each sample data was separated from the off-line data. After the Barcode and primer sequence were intercepted, FLASH (v1.2.7, http://ccb.jhu.edu/software/FLASH/) spliced the reads of each sample (Magoc and Salzberg, 2011), and the splicing sequence obtained was raw tags data (raw tags). Raw tags obtained by splicing need to be strictly filtered to obtain high-quality tags data (clean tags) (Bokulich et al., 2013). Referring to Qiime (v1.9.1, http://qiime.org/scripts/split_libraries_fastq.html), the tag quality control process was as follows (Caporaso et al., 2010). a) tags interception: cut raw tags from the first low-quality base site with a continuous low-quality value (the default quality threshold is < = 19) and a set length (the default length value is 3); b) Tags length filtering: the tags data set obtained by intercepting tags further filters out tags in which the continuous high-quality base length is less than 75% of the tag’s length. The tags obtained after the above processing need to be processed to remove the chimera sequence, and the tags sequence passed Vsearch (https://github.com/torognes/vsearch/) (Rognes et al., 2016) were compared with the species annotation database to detect chimeric sequences, and finally the chimeric sequences were removed to obtain the final effective tags.

The Uparse software (Uparse v7.0.1001, http://www.drive5.com/uparse/) was used to cluster all effective tags of all samples (Haas et al., 2011). By default, the sequence was clustered into OTUs (operational taxonomic units) with 97% identity. At the same time, the representative sequence of OTUs will be selected according to its algorithm principles. The sequence with the highest frequency in OTUs was selected as the representative sequence of OTUs. The bacterial OTUs was detected in Novogene Co.,Ltd. The bacterial diversity index, including OTUs and beta diversity. The beta diversity was represented by the loadings of the first two principal components in the result of PCA (principal components analysis).

### Data analysis

Because the mixed effect models can handle the repeated measurement data, linear mixed-effect models (lmer () function) were utilized to analyzed the effects of nitrogen, month, and year on the soil properties and plant traits. The correlations between repeated measurements within each plot were explicitly accounted for through mixed models. Nitrogen addition, month, and year were set as the fixed factors, and the year was appointed as the random factor. The ls. Means () function was used to compare the means of different treatments. The distance-based redundancy analysis (db-RDA) was employed to test the relationship between soil properties and bacterial diversity.

Using the sem () in package “lavaan”, the SEM model was developed to figure out the direct and indirect factors affecting plant height growth. “soil”, “bacterial”, and “plant” were set as latent variables. The measured variables of “soil” were soil pH, and soil TC, while the measured variables of “bacteria” included bacterial OTUs and beta diversity. The Chi-Square test statistic, CFI (the comparative fit index), SRMR (the standardized root mean square residual), and RMSEA (the root mean square error of approximation) were used to evaluate the goodness of structural equation model fit. The model fits well if it meets the following criteria: (*P*-value of Chi-Square test statistic > 0.05, CFI > 0.95, SRMR < 0.08, and RMSEA < 0.05).

All data were presented as mean ± SE in the text. We deposited the bacterial data in National Library of Medicine (the BioProject ID is PRJNA871067), We performed statistical analyses and drew the figures using in R i386 4.1.1 (R Core Team, 2021) packages “lmerTest”, “lavaan”, “vegan”, and “semPlot”, respectively.

## Results

### The effect of nitrogen addition on soil properties and bacterial compositions

**Figure 1:**
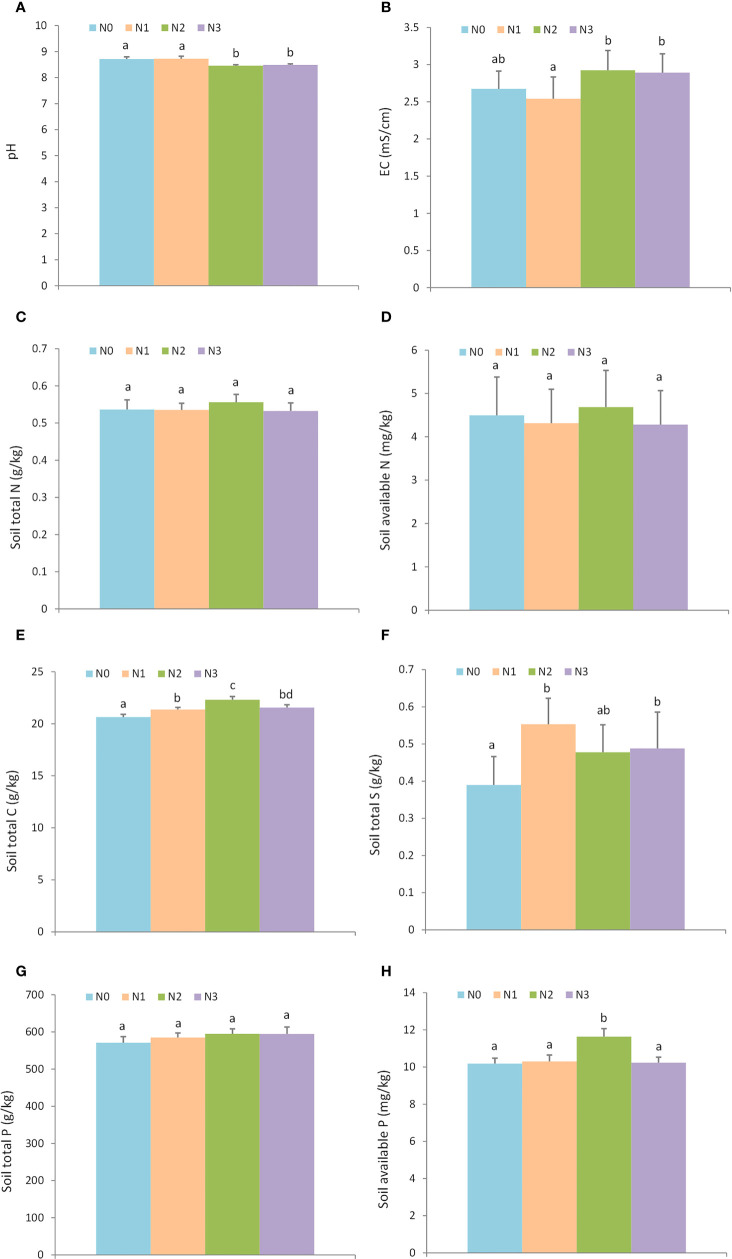
The effect of nitrogen addition on soil physical and chemical properties. N0: 0 g • m^-2^ • year^-1^; N1: 5 g • m^-2^ • year^-1^; N2: 20 g • m^-2^ • year^-1^; N3: 50 g • m^-2^ • year^-1^. Different letters denote statistically significant differences. **A**: pH; **B**: EC (mS/cm); **C**: Soil total N (g/kg); **D**: Soil available N (mg/kg); **E**: Soil total C (g/kg); **F**: Soil total S (g/kg); **G**: Soil total P (g/kg); **H**: Soil available P (mg/kg).

The result showed that nitrogen additions significantly affected soil properties, including pH, salinity, soil TN, soil TS, and soil AP. However, nitrogen additions had little effect on soil total N, soil AN, and soil TP (**Figure 1**, Supporting information **Table S1**). N2 (8.46 ± 0.03) and N3 (8.48 ± 0.04) treatments had lower pH than N0 (8.71 ± 0.09) and N1 (8.72 ± 0.09) (**Figure 1A**), a high level of nitrogen addition increased the soil EC which indicated the salinity level (**Figure 1B**), N2 level quadrats (22.30 ± 0.31 g/kg) had a higher soil TC than N0 (20.65 ± 0.24 g/kg), N1 (21.37 ± 0.20 g/kg), and N3 (21.55 ± 0.27 g/kg) (**Figure 1E**). The nitrogen additions (N1, N2, and N3) increased the soil TS (N0: 0.39 ± 0.08 g/kg, N1: 0.55 ± 0.07 g/kg, N2: 0.48 ± 0.07 g/kg, and N3: 0.48 ± 0.10 g/kg, **Figure 1F**). The level of nitrogen addition N2 resulted in the greatest soil AP (**Figure 1H**).

**Figure 2:**
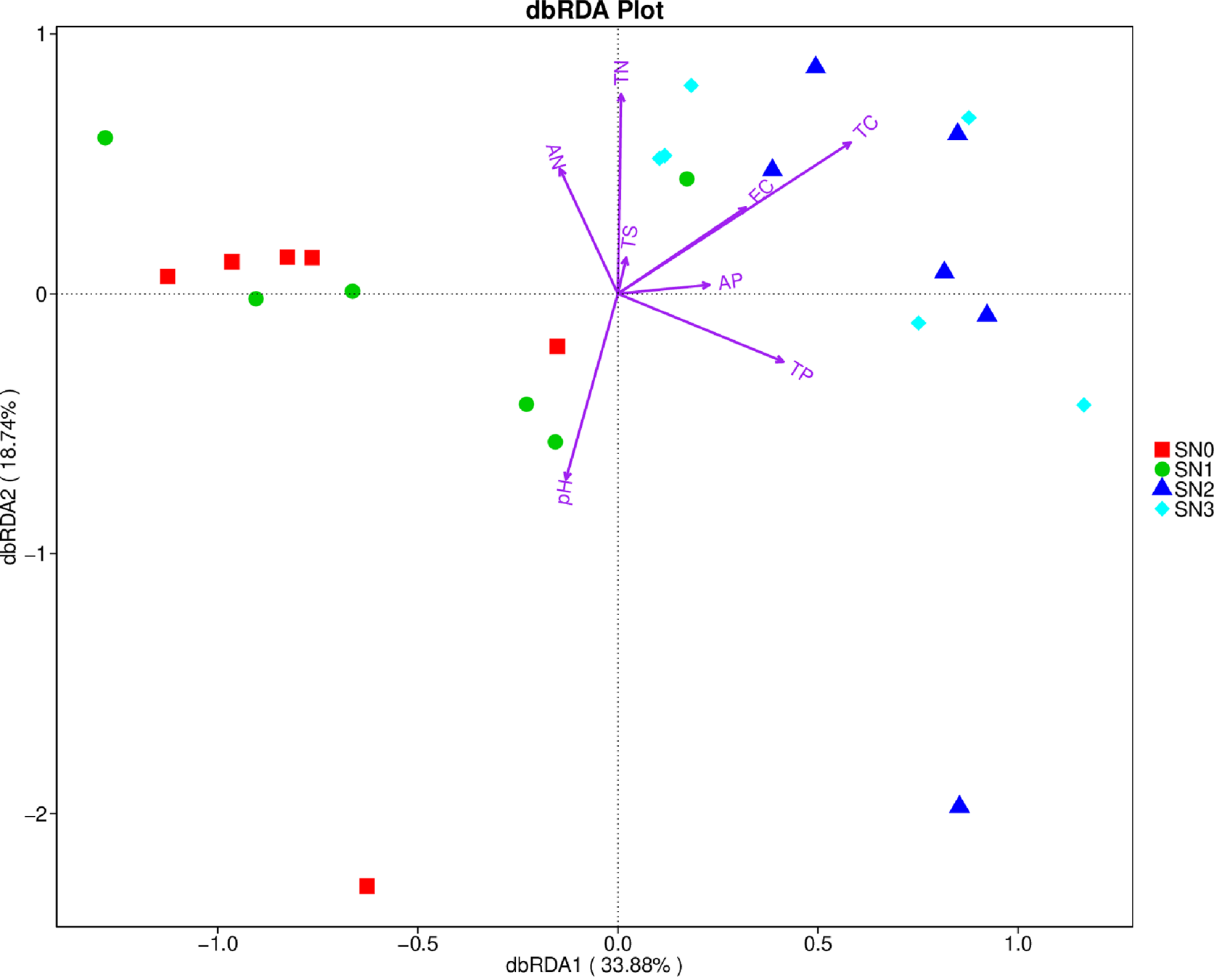
The distance-based redundancy analysis (dbRDA) plot. The relationship of soil physical and chemical properties and bacterial OTUs composition. SN0 is N0 in September 2018, SN1 denotes N1 in September 2018, SN2 means N2 in September 2018 and SN3 indicates N3 in September 2018. AN, soil available N; TN, soil total N; EC, electrical conductivity; TC, soil total N; AP, Soil available P; TP, soil total P; TS, soil total S.

The first two axes of the dbRDA plot accounted for 52.62 percent of the variance in bacterial compositions (OTUs), and the dbRDA plot showed that the most important factors influencing the bacterial compositions were pH (R^2^ = 0.322, *P*-value=0.014), soil TC (R^2^ = 0.415, *P*-value=0.004), soil TN (R^2^ = 0.361, *P*-value=0.006; **Figure 2**; **Table S2**). Soil TC and TN determined the variation in bacterial compositions quadrats of N2 and N3 nitrogen addition levels, whereas pH related to the variation in bacterial compositions of N0 and N1 treatments (**Figure 2**).

### The effect of nitrogen addition on plant traits

**Figure 3:**
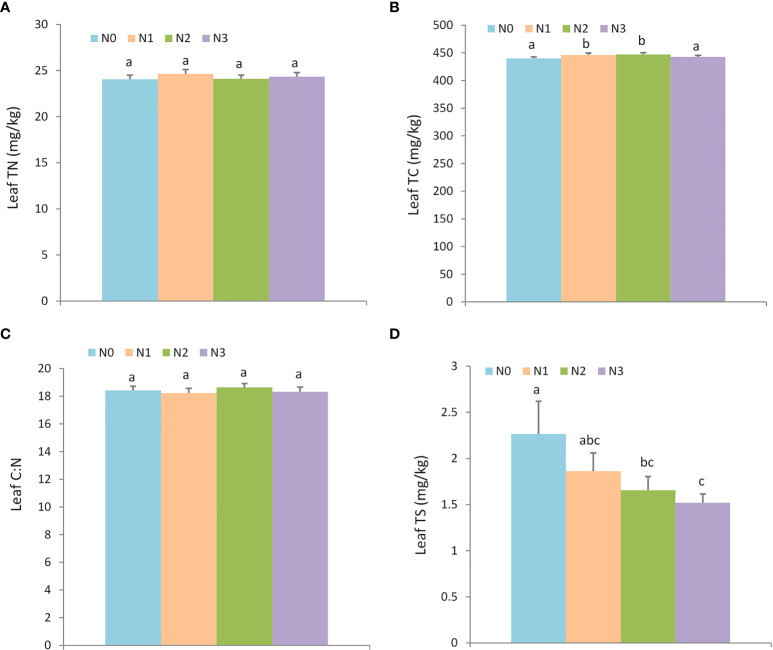
The effect of nitrogen addition on plant nutrients. Different letters denote statistically significant differences. N0: 0 g • m^-2^ • year^-1^; N1: 5 g • m^-2^ • year^-1^; N2: 20 g • m^-2^ • year^-1^; N3: 50 g • m^-2^ • year^-1^. **A**: Leaf TN (mg/kg); **B**: Leaf TC (mg/kg); **C**: Leaf C:N; **D**: Leaf TS (mg/kg).

For the plant nutrient traits, nitrogen addition did not affect the leaf TN and leaf C: N ratio, but it altered the leaf TC and TS (**Figure 3**; **Table S3**). Individuals with N1 (446.08 ± 3.56 g/kg) and N2 (447.18 ± 3.28 g/kg) nitrogen addition levels exhibited higher leaf TC than N0 (439.93 ± 2.52 g/kg) and N3 (442.66 ± 2.71 g/kg), but nitrogen additions decreased the leaf TS compared to the control treatment (N0: 2.26 ± 0.36 g/kg, N1: 1.86 ± 0.20 g/kg, N2: 1.65 ± 0.15 g/kg, and N3: 1.52 ± 0.09 g/kg).

**Figure 4:**
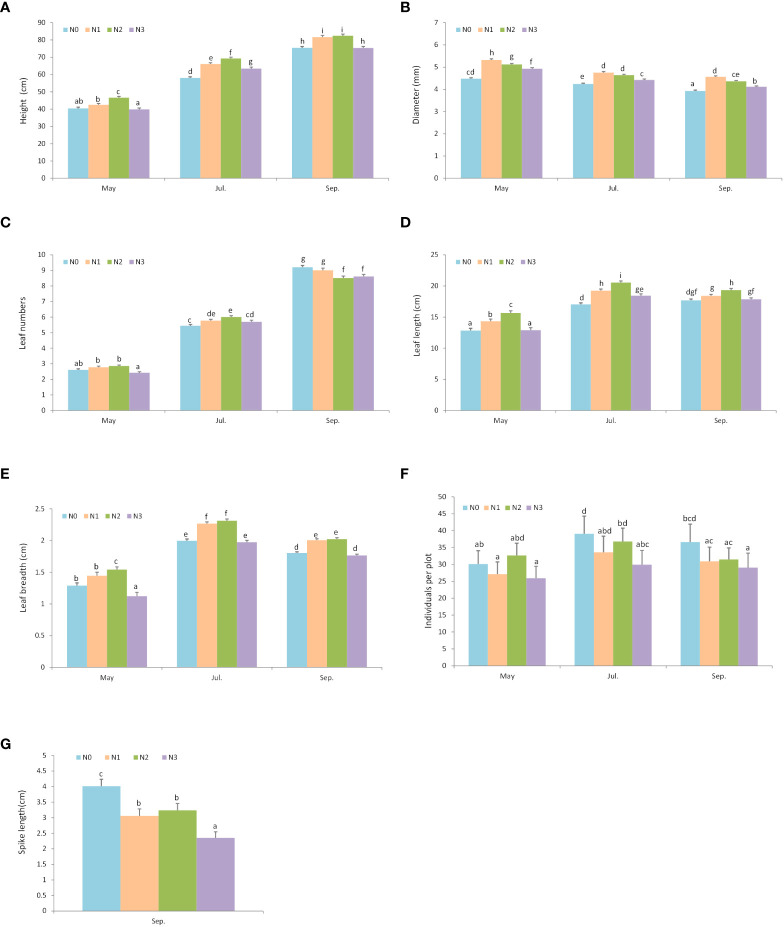
The effect of nitrogen addition on plant growth and reproductive traits in different months. Different letters denote statistically significant differences. N0: 0 g • m^-2^ • year^-1^; N1: 5 g • m^-2^ • year^-1^; N2: 20 g • m^-2^ • year^-1^; N3: 50 g • m^-2^ • year^-1^. **A**: Height; **B**: Diameter (mm); **C**:Leaf numbers; **D**: Leaf length (cm); **E**: Leaf breadth (cm); **F** Individuals per plot; **G**: Spike length (cm).

At the end of the growing season, plants in the N1 and N2 nitrogen addition quadrats (N1: 81.62 ± 0.98 cm; N2: 82.41 ± 0.96 cm) grew taller than those in N0 and N3 quadrats (N0: 75.45 ± 0.76 cm; N3: 75.36 ± 0.89 cm) (**Figure 4A**; **Table 1**). N1, N2, and N3 addition levels promoted diameter growth, but N1 had the greatest diameter excess in May, Jul., and Sep. (**Figure 4B**; **Table S4A**). The effect of nitrogen addition on leaf numbers varied by month (**Figure 4C**; **Table S4B**). In May, N3 reduced the leaf numbers, but N1 and N2 did not significantly differ from N0. In Jul., the leaf number in nitrogen addition treatments (N1, N2, and N3) were significantly greater than N0, and N2 had the greatest leaf numbers. However, in Sep., higher level of nitrogen additions reduced the leaf numbers. The N2 had the greatest leaf length and breadth (**Figures 4D, E**; **Tables S4C, D**). Individuals per plot and spike length decreased as nitrogen addition levels increased (**Figures 4F, G**; **Tables S4E, F**).

**Table 1:** Linear mixed-effects model predicting influences of nitrogen, month (including May, July, and September) on the plant height of the common reed.

Effects	Sum square	Mean square	Num Df	Den Df	F-value	*P*-value
***Fixed effects***						
Nitrogen	9.61×10^4^	3.20×10^4^	3	9.19×10^3^	64.6	<0.001^***^
Month	1.93×10^6^	9.65×10^5^	2	9.19×10^3^	1.94×10^3^	<0.001^***^
Nitrogen: Month	1.42×10^4^	2.37×10^3^	6	9.19×10^3^	4.77	<0.001^***^
***Random effect***						
	**Npar**	**LogLik**	**AIC**	**LRT**	**Df**	***P*-value**
<none>	14	-4.16×10^4^	8.32×10^4^			
(1 |Year)	13	-4.16×10^4^	8.33×10^4^	27.06	1	<0.001^***^

Nitrogen and Month were considered fixed factors; Year was treated as random factors. Npar: number of model parameters; LogLik: the log-likelihood for the model; AIC: the AIC for the model evaluated as -2×(logLik - Npar), and smaller is better; LRT: the likelihood ratio test statistic. ‘^***^’ denotes P-value < 0.001.

### The direct and indirect impacts of nitrogen addition on plant growth

**Figure 5:**
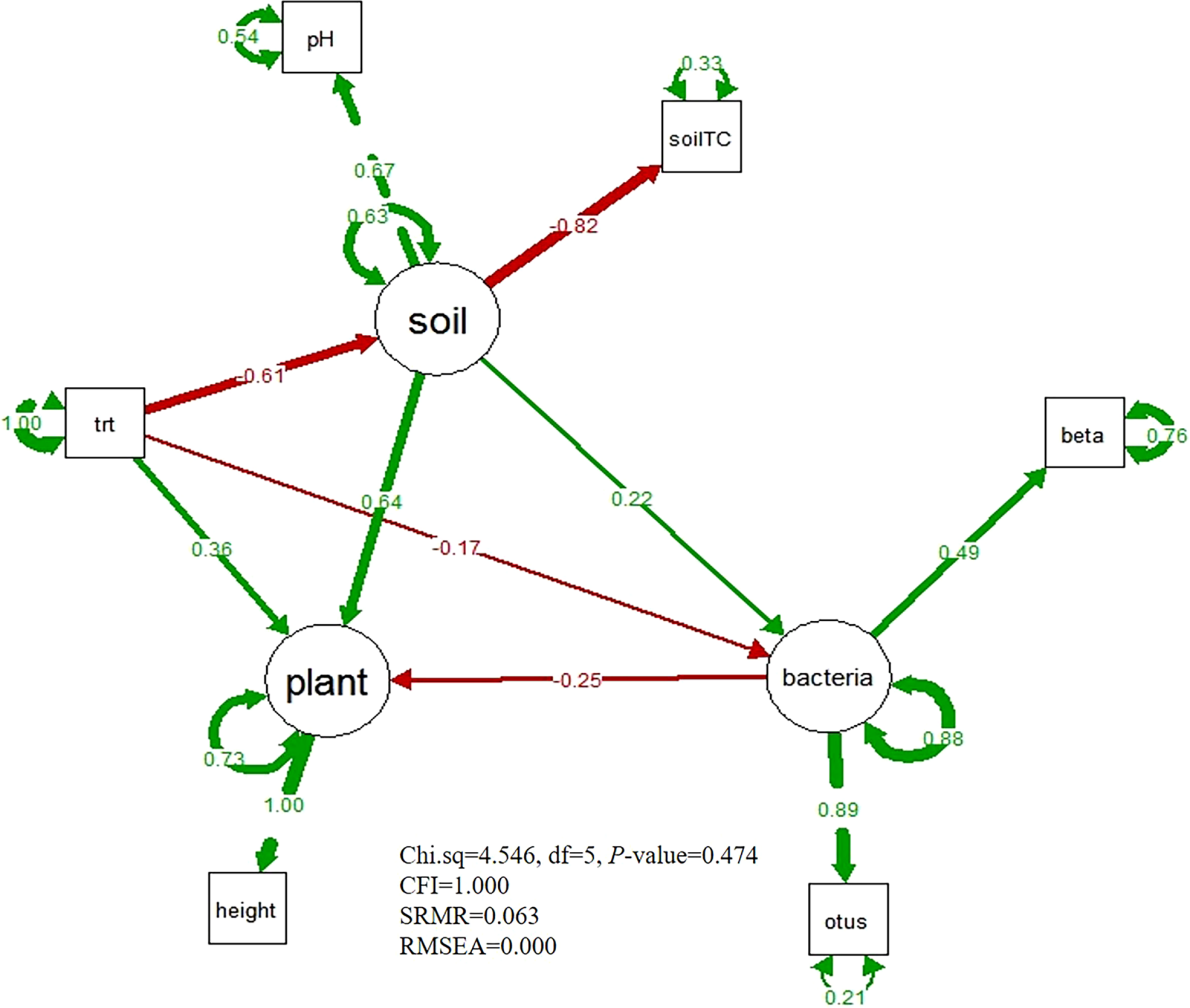
The Structural equation model of nitrogen addition treatment, soil physical and chemical property, bacterial diversity and plant height. The red arrows mean negative effect, the green arrows mean positive effect. Thicker lines denote higher effects. “trt” is the nitrogen addition treatment, “SoilTC” is soil total carbon, “beta” means bacterial beta diversity, “otus” indicates bacterial OTUs.

The goodness of model fit satisfied the SEM model criteria (Chi.sq=4.546, df=5, *P*-value=0.474, CFI=1.000, SRMR=0.063, and RMSEA=0.000). From the SEM model (**Figure 5**), we found that nitrogen addition had a positive direct effect on the plant height (path coefficient=0.36), and soil properties also had a positive direct effect on the plant height (path coefficient=0.64. However, the bacterial diversity had a negatively direct effect on the plant height (path coefficient=-0.25). The soil properties and bacterial diversity were negatively affected by nitrogen enrichment (path coefficient=-0.61; path coefficient=-0.17). By altering bacterial diversity, nitrogen addition had a marginally positive and indirect effect on plant height (path coefficient=0.04). Additionally, nitrogen enrichment also had a small positive impact on the plant height *via* altering both soil properties and bacterial diversity (path coefficient=0.03). However, nitrogen addition decreased plant height through modifying soil properties (path coefficient=-0.39).

## Discussion

### The effect of nitrogen addition on the soil properties in the tidal marsh

Our study was conducted in the tidal marsh, which was flooded frequently by seawater (Zhang et al., 2021). Seawater may reduce the amount of added nitrogen. However, nitrogen addition had a significant impact on soil properties, soil bacterial compositions, and plant growth. Similar to acid soils, nitrogen addition also decreased pH in alkaline soils. This result is inconsistent with study by He et al. (2021b), which revealed that soil pH responded to nitrogen addition modestly in alkaline soil, and nitrogen addition buffered soil acidification by calcium carbonate (Qin et al., 2018). In our study, the pH significantly fell in N3 and N4 compared to N0 and N1, and high nitrogen addition may neutralize the calcium carbonate buffer effect.

The soil salinity did not consistently decrease with increasing of nitrogen addition constantly, and it exhibited a non-linear relationship with nitrogen addition levels. N1 and N0 had lower salinity than N2 and N3. This result is partly consistent with findings of Guan et al. (2019), and they conducted the nitrogen addition experiment in the non-tidal coastal wetland of YRD and found that salinity had a negative relationship with nitrogen addition. This experiment had a lower nitrogen addition rate than our work, and the nitrogen substance used in the experiment was NH_4_NO_3_ (we used urea) which may immediately increase soil NH_4_^+^ and NO_3_^-^ after nitrogen addition. Because the association of soil exchangeable base cations and soil NH_4_^+^ and NO_3_^-^ was negative, salinity decreased with nitrogen addition (Qin et al., 2018).

According to our study, neither soil TN nor AN changed after nitrogen addition. This result may have been caused by three factors. Initially, plants and microorganisms absorbed and consumed nitrogen, thereby reducing the amount of added nitrogen. Secondly, the seawater may have washed away some fertilizer, but the amount of fertilizer washed away has not yet been quantified. Thirdly, a portion of the urea had not been converted into inorganic nitrogen but had instead remained in organic form.

In accordance with findings from other studies, nitrogen addition increased soil TC (Guan et al., 2019; Sun et al., 2020). The primary reason may be that nitrogen addition increased the productivity of plants and microbes, resulting in a greater return of litter or microbial carbon to the soil. The highest soil AP was found in N2, which may be associated with the decomposition rate of plants and microorganisms. However, the highest nitrogen addition (N3) rate inhibited phosphatase and consequently decreased soil organic P mineralization (Olander and Vitousek, 2000). Few studies have examined the effect of nitrogen addition on soil S. Sulfur is an essential element in the coastal wetland, and its influence on plant growth depends on the chemical species present (sulfate or sulfide). Intriguingly, nitrogen addition increased soil TS. A previous study demonstrated that nitrogen addition can inhibit the activity of soil arylsulfatase due to a decrease in pH, which could mineralize the organic S into inorganic form (Chen et al., 2016a). Wang et al. (2016) found that nitrogen addition to the grassland had no effect on soil TS but increased soil available S. The outcome of the grassland differs from that of the coastal wetland. The mechanism underlying the response of coastal wetland soil TS to nitrogen addition requires further investigation.

### The effect of nitrogen addition on bacterial compositions and plant traits in the tidal marsh

Nitrogen addition altered soil physical and chemical factors, and bacterial compositions was also affected by environmental variables. Significantly influenced by nitrogen addition, soil pH and TC played critical roles in the variation of soil bacterial compositions. This result is consistent with previous studies. Soil acidification caused by nitrogen addition can reduce bacterial, fungal, and actinobacteria biomass (Chen et al., 2015) as well as bacterial diversity (Zhou et al., 2022). In a non-tidal coastal wetland nitrogen addition experiment, Lu et al. (2021) also found that soil TC was one of the most crucial factors influencing soil bacterial diversity. Thus, soil acidification and resource availability contributed to the altered bacterial composition following nitrogen addition.

The leaf TC and TS also changed as a result of nitrogen addition. The moderate nitrogen addition level promoted carbon assimilation in plant leaves, but the positive effect disappeared at higher levels. It suggests that excessive nitrogen addition can inhibit the carbon dioxide adsorption or organic carbon synthesis, thereby suppressing plant growth. Sun et al. (2020) and Jing et al. (2017) revealed that nitrogen addition increased the carbon content of plant leaves. While the leaf TS decreased with nitrogen addition, this suggests that plants absorb less S as nitrogen addition levels increase. There are two possible causes for this outcome. Firstly, the decrease of inorganic S due to a decrease in soil arylsulfatase activity in higher nitrogen addition levels. Secondly, the plant allocated fewer resources for S acquisition because it must allocate more resources for P acquisition, which is the first limited resource (Chen et al., 2016a). It contradicts the findings of Li et al. (2019), and they discovered that plant S uptake increased with nitrogen addition in a S-deficient temperate steppe. This discrepancy may be caused by varying S supply levels.

Furthermore, our study revealed the linkages between nitrogen addition, soil environment, bacterial diversity, and plant growth in the coastal wetland. Few studies have investigated these connections in the coastal wetland ecosystems. Our study may partially fill this knowledge gap. In a coastal wetland, nitrogen addition had a direct and positive effect on plant growth, and a positive or negative indirect effect on aboveground plant growth, primarily by altering soil properties (i.e., soil pH and TC), and bacterial composition. Totally, the indirect effect contributed similarly but contrarily to the direct effect. The direct effect is the result of the added nitrogen being utilized by plants. Liu et al. (2020) demonstrated that nitrogen addition increased plant aboveground biomass directly in the grassland, but they did not investigate the effect of soil property and bacterial richness on plant growth. Nevertheless, numerous studies have documented the substantial effect of plant growth or diversity resulting from nitrogen addition on soil properties and the bacterial community (Li et al., 2013; Zeng et al., 2016; Liu et al., 2020). Consequently, the bi-directional interactions between plant community, soil properties and bacterial community is crucial for the response of ecosystem structure to nitrogen deposition.

Finally, in the tidal marsh, even at the lowest input level (5 g • m^-2^ • year^-1^), the nitrogen addition had a significant effect on soil physical and chemical properties, soil bacterial compositions, and plant growth despite the flush of the sea tide. At the maximum level of nitrogen input, plant growth was inhibited. Consider the tidal flush, when nitrogen supply greater than 20 g • m^-2^ • year^-1^ and less than 50 g • m^-2^ • year^-1^ inhibited plant growth. Therefore, under the scenario of future high nitrogen inputs, the growth of coastal wetland vegetation, including tidal marshes, will be hampered. It is suggested that the regulation of agricultural nitrogen inputs is still necessary to protect vegetation.

## Conclusion

The addition of nitrogen significantly altered pH, salinity, soil TC and soil TS in the coastal tidal marsh. Soil pH and TC were the most influential environmental influences on the soil bacterial compositions. Intermediate nitrogen addition enhanced plant growth and reproductive traits significantly, with N1 and N2 having greater values for these traits than N0 and N3. Nitrogen addition influenced plant height in the coastal wetland both directly and indirectly, and the indirect but negative effect was mainly caused by soil properties change induced by nitrogen addition. Additional research is required to investigate different ecological functions of the coastal wetland under future nitrogen input scenarios.

## Data availability statement

The original contributions presented in the study are publicly available. This data can be found here: NCBI, PRJNA871067, available at https://www.ncbi.nlm.nih.gov/bioproject/PRJNA871067.

## Author contributions

LWZ: Conceptualization, Methodology, Investigation, Data curation, Visualization, Writing Original draft preparation, and Funding acquisition. LJZ, HPY, SQL & LC: Methodology and Investigation. GXH: Conceptualization and Supervision. All authors contributed to the article and approved the submitted version.

## Funding

This study is supported by grants from the National Natural Science Foundation of China (31971504 and 31670533), and the Youth Innovation Promotion Association CAS (2018247).

## Conflict of interest

The authors declare that the research was conducted in the absence of any commercial or financial relationships that could be construed as a potential conflict of interest.

## Publisher’s note

All claims expressed in this article are solely those of the authors and do not necessarily represent those of their affiliated organizations, or those of the publisher, the editors and the reviewers. Any product that may be evaluated in this article, or claim that may be made by its manufacturer, is not guaranteed or endorsed by the publisher.
