# Supplementary material for: Nitrogen addition alters plant growth in China’s Yellow River Delta coastal wetland through direct and indirect effects
Source: Front Plant Sci. 2022 Oct 13;13:1016949. doi: 10.3389/fpls.2022.1016949 (PMC9606764; doi:10.3389/fpls.2022.1016949)
Supplement: Supplementary file 1 [file DataSheet_1.docx]

**Supporting information**

**Table S1** Linear mixed-effects model predicting influences of nitrogen addition on the soil physical and chemical properties.

(a) pH

| **Effects** | **Sum square** | **Mean square** | **Num Df** | **Den Df** | **F-value** | ***P*-value** |
| --- | --- | --- | --- | --- | --- | --- |
| ***Fixed effects*** |  |  |  |  |  |  |
| Nitrogen | 1.48 | 0.49 | 3 | 89 | 13.63 | <0.001^***^ |
| ***Random effect*** |  |  |  |  |  |  |
|  | **Npar** | **LogLik** | **AIC** | **LRT** | **Df** | ***P*-value** |
| <none> | 6 | 9.45 | -6.91 |  |  |  |
| (1 \|Year) | 5 | -39.74 | 89.48 | 98.39 | 1 | <0.001^***^ |

(b) Salinity

| **Effects** | **Sum square** | **Mean square** | **Num Df** | **Den Df** | **F-value** | ***P*-value** |
| --- | --- | --- | --- | --- | --- | --- |
| ***Fixed effects*** |  |  |  |  |  |  |
| Nitrogen | 2.40 | 0.80 | 3 | 89 | 3.65 | 0.016^**^ |
| ***Random effect*** |  |  |  |  |  |  |
|  | **Npar** | **LogLik** | **AIC** | **LRT** | **Df** | ***P*-value** |
| <none> | 6 | -75.04 | 162.1 |  |  |  |
| (1 \|Year) | 5 | -160.1 | 330.2 | 170.1 | 1 | <0.001^***^ |

(c) Soil available N

| **Effects** | **Sum square** | **Mean square** | **Num Df** | **Den Df** | **F-value** | ***P*-value** |
| --- | --- | --- | --- | --- | --- | --- |
| ***Fixed effects*** |  |  |  |  |  |  |
| Nitrogen | 2.49 | 0.83 | 3 | 89 | 0.69 | 0.56 |
| ***Random effect*** |  |  |  |  |  |  |
|  | **Npar** | **LogLik** | **AIC** | **LRT** | **Df** | ***P*-value** |
| <none> | 6 | -154.6 | 321.3 |  |  |  |
| (1 \|Year) | 5 | -265.7 | 541.3 | 222.1 | 1 | <0.001^***^ |

(d) Soil total N

| **Effects** | **Sum square** | **Mean square** | **Num Df** | **Den Df** | **F-value** | ***P*-value** |
| --- | --- | --- | --- | --- | --- | --- |
| ***Fixed effects*** |  |  |  |  |  |  |
| Nitrogen | 8.56×10^-3^ | 2.85×10^-3^ | 3 | 89 | 0.53 | 0.67 |
| ***Random effect*** |  |  |  |  |  |  |
|  | **Npar** | **LogLik** | **AIC** | **LRT** | **Df** | ***P*-value** |
| <none> | 6 | 97.73 | -183.5 |  |  |  |
| (1 \|Year) | 5 | 69.38 | -128.8 | 56.70 | 1 | <0.001^***^ |

(f) Soil total C

| **Effects** | **Sum square** | **Mean square** | **Num Df** | **Den Df** | **F-value** | ***P*-value** |
| --- | --- | --- | --- | --- | --- | --- |
| ***Fixed effects*** |  |  |  |  |  |  |
| Nitrogen | 33.11 | 11.04 | 3 | 89 | 8.02 | <0.001^***^ |
| ***Random effect*** |  |  |  |  |  |  |
|  | **Npar** | **LogLik** | **AIC** | **LRT** | **Df** | ***P*-value** |
| <none> | 6 | -154.3 | 320.7 |  |  |  |
| (1 \|Year) | 5 | -158.8 | 327.5 | 8.87 | 1 | 0.003^**^ |

(g) Soil total S

| **Effects** | **Sum square** | **Mean square** | **Num Df** | **Den Df** | **F-value** | ***P*-value** |
| --- | --- | --- | --- | --- | --- | --- |
| ***Fixed effects*** |  |  |  |  |  |  |
| Nitrogen | 0.24 | 0.08 | 3 | 66 | 4.04 | 0.011^*^ |
| ***Random effect*** |  |  |  |  |  |  |
|  | **Npar** | **LogLik** | **AIC** | **LRT** | **Df** | ***P*-value** |
| <none> | 6 | 25.51 | -39.03 |  |  |  |
| (1 \|Year) | 5 | -29.01 | 68.02 | 109.1 | 1 | <0.001^***^ |

(h) Soil total P

| **Effects** | **Sum square** | **Mean square** | **Num Df** | **Den Df** | **F-value** | ***P*-value** |
| --- | --- | --- | --- | --- | --- | --- |
| ***Fixed effects*** |  |  |  |  |  |  |
| Nitrogen | 6.70×10^3^ | 2.23×10^3^ | 3 | 66 | 1.67 | 0.182 |
| ***Random effect*** |  |  |  |  |  |  |
|  | **Npar** | **LogLik** | **AIC** | **LRT** | **Df** | ***P*-value** |
| <none> | 6 | -351.3 | 714.7 |  |  |  |
| (1 \|Year) | 5 | -386.4 | 782.7 | 70.06 | 1 | <0.001^***^ |

(i) Soil available P

| **Effects** | **Sum square** | **Mean square** | **Num Df** | **Den Df** | **F-value** | ***P*-value** |
| --- | --- | --- | --- | --- | --- | --- |
| ***Fixed effects*** |  |  |  |  |  |  |
| Nitrogen | 26.22 | 8.74 | 3 | 66 | 6.90 | <0.001^***^ |
| ***Random effect*** |  |  |  |  |  |  |
|  | **Npar** | **LogLik** | **AIC** | **LRT** | **Df** | ***P*-value** |
| <none> | 6 | -113.5 | 239.0 |  |  |  |
| (1 \|Year) | 5 | -127.9 | 265.7 | 28.71 | 1 | <0.001^***^ |

Note: Nitrogen and Month were considered fixed factors; Year was treated as random factors. Npar: number of model parameters; LogLik: the log-likelihood for the model; AIC: the AIC for the model evaluated as -2×(logLik - Npar), and smaller is better; LRT: the likelihood ratio test statistic. ‘^***^’ denotes *P*-value<0.001; ‘^**^’ denotes *P*-value<0.01; ‘^*^’ indicates *P*-value<0.05.

**Table S2** The dbRDA ordination of soil factors and bacterial OTUs. ‘^**^’ indicates *P*-value <0.01; ‘^*^’ indicates *P*-value<0.05.

| **Soil factors** | **dbRDA1** | **dbRDA2** | **R^2^** | **P-value** |
| --- | --- | --- | --- | --- |
| pH | -0.167 | -0.986 | 0.322 | 0.014^*^ |
| EC | 0.706 | 0.708 | 0.130 | 0.229 |
| Soil available N | -0.314 | 0.949 | 0.157 | 0.155 |
| Soil total N | -0.009 | 1.000 | 0.361 | 0.006^**^ |
| Soil total C | 0.720 | 0.694 | 0.415 | 0.004^**^ |
| Soil total S | 0.136 | 0.991 | 0.012 | 0.881 |
| Soil total P | 0.849 | -0.528 | 0.154 | 0.165 |
| Soil available P | 0.992 | 0.124 | 0.034 | 0.689 |

**Table S3** Linear mixed-effects model predicting influences of nitrogen on the leaf nutrients.

(a) Leaf total N

| **Effects** | **Sum square** | **Mean square** | **Num Df** | **Den Df** | **F-value** | **P-value** |
| --- | --- | --- | --- | --- | --- | --- |
| ***Fixed effects*** |  |  |  |  |  |  |
| Nitrogen | 4.91 | 1.64 | 3 | 86.01 | 0.42 | 0.74 |
| ***Random effect*** |  |  |  |  |  |  |
|  | **Npar** | **LogLik** | **AIC** | **LRT** | **Df** | **P-value** |
| <none> | 6 | -195.9 | 403.8 |  |  |  |
| (1 \|Year) | 5 | -199.6 | 409.2 | 7.38 | 1 | 0.007^**^ |

(b) Leaf total C

| **Effects** | **Sum square** | **Mean square** | **Num Df** | **Den Df** | **F-value** | **P-value** |
| --- | --- | --- | --- | --- | --- | --- |
| ***Fixed effects*** |  |  |  |  |  |  |
| Nitrogen | 747.1 | 249.0 | 3 | 86.0 | 3.31 | 0.024^*^ |
| ***Random effect*** |  |  |  |  |  |  |
|  | **Npar** | **LogLik** | **AIC** | **LRT** | **Df** | **P-value** |
| <none> | 6 | -330.8 | 673.6 |  |  |  |
| (1 \|Year) | 5 | -371.3 | 752.6 | 81.1 | 1 | <0.001^***^ |

(c) Leaf C:N

| **Effects** | **Sum square** | **Mean square** | **Num Df** | **Den Df** | **F-value** | **P-value** |
| --- | --- | --- | --- | --- | --- | --- |
| ***Fixed effects*** |  |  |  |  |  |  |
| Nitrogen | 1.88 | 0.63 | 3 | 86.0 | 0.30 | 0.83 |
| ***Random effect*** |  |  |  |  |  |  |
|  | **Npar** | **LogLik** | **AIC** | **LRT** | **Df** | **P-value** |
| <none> | 6 | -168.1 | 348.2 |  |  |  |
| (1 \|Year) | 5 | -170.5 | 350.1 | 4.73 | 1 | 0.03^*^ |

(d) Leaf total S

| **Effects** | **Sum square** | **Mean square** | **Num Df** | **Den Df** | **F-value** | **P-value** |
| --- | --- | --- | --- | --- | --- | --- |
| ***Fixed effects*** |  |  |  |  |  |  |
| Nitrogen | 5.70 | 1.90 | 3 | 66 | 3.81 | 0.014^*^ |
| ***Random effect*** |  |  |  |  |  |  |
|  | **Npar** | **LogLik** | **AIC** | **LRT** | **Df** | **P-value** |
| <none> | 6 | -81.9 | 175.8 |  |  |  |
| (1 \|Year) | 5 | -98.1 | 206.3 | 32.45 | 1 | <0.001^***^ |

Note: Nitrogen and Month were considered fixed factors; Year was treated as random factors. Npar: number of model parameters; LogLik: the log-likelihood for the model; AIC: the AIC for the model evaluated as -2×(logLik - Npar), and smaller is better; LRT: the likelihood ratio test statistic; ‘^***^’ denotes P-value<0.001; ‘^**^’ indicates P-value <0.01; ‘^*^’ indicates P-value<0.05.

**Table S4** Linear mixed-effects model predicting influences of nitrogen, month (including June, July, August and September) on the plant traits of the common reed.

(a) Diameter

| **Effects** | **Sum square** | **Mean square** | **Num Df** | **Den Df** | **F-value** | ***P*-value** |
| --- | --- | --- | --- | --- | --- | --- |
| ***Fixed effects*** |  |  |  |  |  |  |
| Nitrogen | 363.4 | 121.1 | 3 | 5.36×10^3^ | 170.3 | <0.001^***^ |
| Month | 469.9 | 234.9 | 2 | 5.36×10^3^ | 330.2 | <0.001^***^ |
| Nitrogen: Month | 16.2 | 2.70 | 6 | 5.36×10^3^ | 3.80 | <0.001^***^ |
| ***Random effect*** |  |  |  |  |  |  |
|  | **Npar** | **LogLik** | **AIC** | **LRT** | **Df** | ***P*-value** |
| <none> | 14 | -6.73×10^3^ | 1.35×10^4^ |  |  |  |
| (1 \|Year) | 13 | -6.84×10^3^ | 1.37×10^4^ | 219.7 | 1 | <0.001^***^ |

(b) Leaf numbers

| **Effects** | **Sum square** | **Mean square** | **Num Df** | **Den Df** | **F-value** | ***P*-value** |
| --- | --- | --- | --- | --- | --- | --- |
| ***Fixed effects*** |  |  |  |  |  |  |
| Nitrogen | 80 | 26.5 | 3 | 9.19×10^3^ | 3.54 | 0.014^*^ |
| Month | 5.51×10^4^ | 2.76×10^4^ | 2 | 9.19×10^3^ | 3.67×10^3^ | <0.001^***^ |
| Nitrogen: Month | 383 | 63.9 | 6 | 9.19×10^3^ | 8.51 | <0.001^***^ |
| ***Random effect*** |  |  |  |  |  |  |
|  | **Npar** | **LogLik** | **AIC** | **LRT** | **Df** | ***P*-value** |
| <none> | 14 | -2.23×10^4^ | 4.47×10^4^ |  |  |  |
| (1 \|Year) | 13 | -2.23×10^4^ | 4.47×10^4^ | 45.46 | 1 | <0.001^***^ |

(c) Leaf length

| **Effects** | **Sum square** | **Mean square** | **Num Df** | **Den Df** | **F-value** | ***P*-value** |
| --- | --- | --- | --- | --- | --- | --- |
| ***Fixed effects*** |  |  |  |  |  |  |
| Nitrogen | 1.01×10^4^ | 3.36×10^3^ | 3 | 9.18×10^3^ | 54.35 | <0.001^***^ |
| Month | 4.15×10^4^ | 2.07×10^4^ | 2 | 9.19×10^3^ | 334.9 | <0.001^***^ |
| Nitrogen: Month | 1.06×10^3^ | 176.6 | 6 | 9.19×10^3^ | 2.85 | 0.009^**^ |
| ***Random effect*** |  |  |  |  |  |  |
|  | **Npar** | **LogLik** | **AIC** | **LRT** | **Df** | ***P*-value** |
| <none> | 14 | -3.20×10^4^ | 6.41×10^4^ |  |  |  |
| (1 \|Year) | 13 | -3.20×10^4^ | 6.41×10^4^ | 5.84 | 1 | 0.016^**^ |

(d) Leaf breadth

| **Effects** | **Sum square** | **Mean square** | **Num Df** | **Den Df** | **F-value** | ***P*-value** |
| --- | --- | --- | --- | --- | --- | --- |
| ***Fixed effects*** |  |  |  |  |  |  |
| Nitrogen | 142.1 | 47.4 | 3 | 8.04×10^3^ | 79.6 | <0.001^***^ |
| Month | 611.8 | 305.9 | 2 | 6.59×10^3^ | 514.4 | <0.001^***^ |
| Nitrogen: Month | 6.54 | 1.09 | 6 | 8.05×10^3^ | 1.83 | 0.09 |
| ***Random effect*** |  |  |  |  |  |  |
|  | **Npar** | **LogLik** | **AIC** | **LRT** | **Df** | ***P*-value** |
| <none> | 14 | -9.37×10^4^ | 1.88×10^4^ |  |  |  |
| (1 \|Year) | 13 | -9.37×10^4^ | 1.88×10^4^ | 9.43 | 1 | 0.002^**^ |

(e) Individuals

| **Effects** | **Sum square** | **Mean square** | **Num Df** | **Den Df** | **F-value** | ***P*-value** |
| --- | --- | --- | --- | --- | --- | --- |
| ***Fixed effects*** |  |  |  |  |  |  |
| Nitrogen | 1.67×10^3^ | 834.8 | 2 | 273 | 3.39 | 0.035^*^ |
| Month | 2.10×10^3^ | 700.8 | 3 | 273 | 2.84 | 0.038^*^ |
| Nitrogen: Month | 455.3 | 75.89 | 6 | 273 | 0.31 | 0.93 |
| ***Random effect*** |  |  |  |  |  |  |
|  | **Npar** | **LogLik** | **AIC** | **LRT** | **Df** | ***P*-value** |
| <none> | 14 | -1.18×10^3^ | 2.38×10^3^ |  |  |  |
| (1 \|Year) | 13 | -1.25×10^3^ | 2.52×10^3^ | 138.8 | 1 | <0.001^***^ |

(f) Spike length

| **Effects** | **Sum square** | **Mean square** | **Num Df** | **Den Df** | **F-value** | ***P*-value** |
| --- | --- | --- | --- | --- | --- | --- |
| ***Fixed effects*** |  |  |  |  |  |  |
| Nitrogen | 1.20×10^3^ | 397.6 | 3 | 3.07×10^3^ | 12.0 | <0.001^***^ |
| ***Random effect*** |  |  |  |  |  |  |
|  | **Npar** | **LogLik** | **AIC** | **LRT** | **Df** | ***P*-value** |
| <none> | 6 | -9.77×10^3^ | 1.95×10^4^ |  |  |  |
| (1 \|Year) | 5 | -9.87×10^3^ | 1.97×10^4^ | 210.5 | 1 | <0.001^***^ |

Note: Nitrogen and Month were considered fixed factors; Year was treated as random factors. Npar: number of model parameters; LogLik: the log-likelihood for the model; AIC: the AIC for the model evaluated as -2×(logLik - Npar), and smaller is better; LRT: the likelihood ratio test statistic. ‘^***^’ denotes P-value<0.001; ‘^*^’ indicates *P*-value<0.05.
